# Oxysterol 25-hydroxycholesterol activation of ferritinophagy inhibits the development of squamous intraepithelial lesion of cervix in HPV-positive patients

**DOI:** 10.1038/s41420-024-01899-3

**Published:** 2024-03-13

**Authors:** Tianming Wang, Min Gong, Yingfei Lu, Chengcheng Zhao, Ling Ling, Jianquan Chen, Rong Ju

**Affiliations:** 1 Central Laboratory, Translational Medicine Research Center, The Affiliated Jiangning Hospital with Nanjing Medical University, Nanjing, Jiangsu China; 2 Department of Obstetrics and Gynecology, The Affiliated Jiangning Hospital with Nanjing Medical University, Nanjing, Jiangsu China

**Keywords:** Cervical cancer, Oncogenesis

## Abstract

Squamous intraepithelial lesion of cervix (SIL) in human papillomavirus (HPV)-positive patient often undergoes a silent and long-course development, and most of them with high-grade transit to cervical squamous cell carcinoma (CSCC). The oxysterol 25-hydroxycholesterol (25-HC) is associated with HPV inhibition, autophagy and cholesterol synthesis, however, its function in this long process of SIL development remain unclear. In this study, we demonstrate that 25-HC generation is inhibited through HSIL-to-CSCC transition. The 25-HC activates ferritinophagy in the early stage of SIL, promoting the vulnerability of HSILs to ferroptosis. Therefore, maintaining 25-HC level is crucial for suppressing HSIL progression and holds promise for developing novel clinical therapies for CSCC.

## Introduction

Cervical cancer is one of the most common cancers, affecting over half a million women across the globe [1]. One of the main subtypes of this cancer is cervical squamous cell carcinoma (CSCC), which accounts for 70% of patients [2]. Due to its inconspicuous early onset and prolonged course, understanding the molecular mechanism of CSCC is crucial for diagnosis and clinical therapy. The emergence and development of CSCC are associated with high-risk subtypes of human papillomavirus (HPV) infection, including HPV16 [3–5]. Persistent HPV infection leads to low-grade squamous intraepithelial lesion (LSIL), a portion of LSILs eventually develop into high-grade squamous intraepithelial lesion (HSIL), and most HSILs further convert to CSCC [6]. Previous studies have indicated that cholesterol metabolites known as oxysterols have the ability to inhibit the replication of human viral pathogens [7]. One specific type of oxysterols, called 25-hydroxycholesterol (25-HC), is produced by an enzyme called cholesterol-25-hydroxylase (CH25H) [8]. 25-HC has broad antiviral properties, effectively inhibiting the replication of enveloped and non-enveloped viruses, including HPV16, HRoV, and HRhV [9, 10]. However, the roles of endogenous 25-HC in the emergence and development of LSIL, HSIL, and CSCC have been rarely studied.

Ferroptosis is a type of regulated cell death that leads to oxidative damage, characterized by the accumulation of Fe (II) and increased lipid peroxidation [11]. With the help of a transmission electron microscope, the rupture of outer mitochondrial membrane density and the reduction of mitochondrial inner cristae partly reflect the ultramicroscopic characteristics of ferroptosis [12]. Among the various pathways associated with ferroptosis, ferritinophagy displays an important manner for ferritin-iron release [13]. In ferritinophagy, transferrin receptor protein 1 (TFRC) functions as iron intake and intracellular transport, nuclear receptor coactivator 4 (NCOA4) delivers ferritin-iron complex to lysosomes for iron release, and the activation of autophagy is also required [13, 14]. Intracellular cholesterol homeostasis maintenance is closely linked to ferroptosis. Abnormal activation of the mevalonate pathway in cholesterol synthesis results in an increase of isopentenyl pyrophosphate, which inhibits ferroptosis by upregulating the expression of glutathione peroxidase 4 (GPX4) [15]. Recently, 25-HC has been reported to induce oxiapoptophagy in a mouse fibroblast cell line [16]. However, the impact of intracellular iron homeostasis maintenance related to 25-HC in CSCC has rarely been investigated. Our previous study demonstrated that ferroptosis occurred in SIL stage but anti-ferroptosis emerged during the CSCC stage [17]. Based on this, we hypothesized that 25-HC might regulate ferroptosis and play a role in the progress of the HSIL-to-CSCC transition.

In this study, we assessed the effect of 25-HC on the regulation of intracellular iron levels, investigated its underlying mechanism in vitro and in vivo. Our findings revealed that 25-HC facilitated ferritinophagy during the SIL stage, and that 25-HC generation was reduced through SIL-to-CSCC transition. Overall, our study provides crucial insights into the role of 25-HC in the occurrence of CSCC and strongly implies its potential for developing novel clinical therapies for CSCC.

## Results

### Endogenous oxysterol 25-HC is at high level in HSILs

**Fig. 1:**
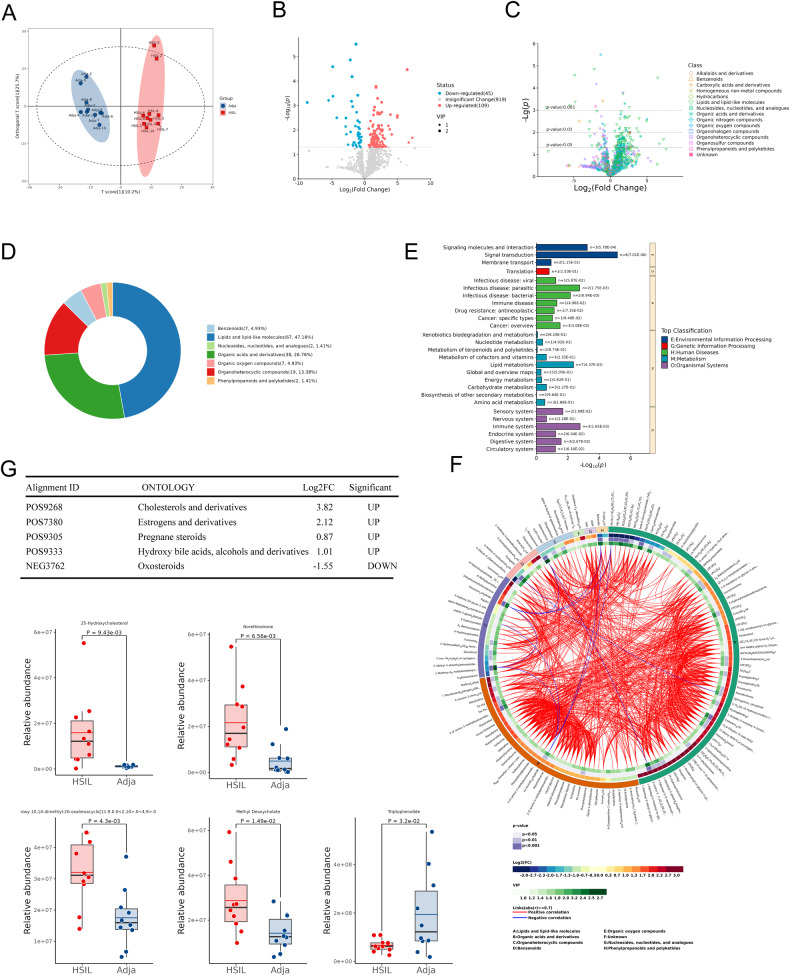
Differential metabolites between HSIL and adjacent tissues are screened via metabolomics analysis. **A** OPLS-DA of metabolites profiles of HSIL and adjacent tissues (*n* = 10). **B** Metabolites data was used to identify differential metabolites by Volcano plot. Red and blue dots respectively indicated the upregulated and downregulated metabolites. **C**, **D** Volcano plot and ring diagram were used to classified differential metabolites data with HMDB superclass. **E** Differential metabolites in HSIL vs adjacent tissues enriched in pathway were shown in KEGG analysis. The abscissa represents the metabolites number. **F** Circos-diagram was used to show the differential metabolites in classification, abundance of each group, VIP score and correlation of each metabolite. Fold change data was normalized by logarithm of 2. Red and blue lines, respectively, indicated the positive and negative correlation. **G** Differential metabolite abundance analysis was used to show the relative abundance of candidate metabolites.

In the process of tumorigenesis, cells often undergo metabolic reprogramming to adapt to changes in their microenvironment [18], so we first performed a mass spectrometry-based metabolomics approaches to detect and quantify metabolites in HSIL tissues. There were 10 cases of HSIL tissues with their adjacent tissues collected from patients undergoing cervical conization. Orthogonal partial least squares-discriminant analysis (OPLS-DA) showed that the metabolites data, which was standardized and spread across the panel, showed distinct differences in metabolite between HSIL and adjacent tissues (Fig. 1A). The significant metabolites changes were detected with 154 (14.35%) differentially expressed metabolites out of 1073 detectable metabolites (|log2FC| > 1 and *p* < 0.05), with 109 upregulated and 45 downregulated metabolites (Fig. 1A–C). Based on the structure and function of metabolites, the differential metabolites were classified and statistically analyzed. According to the ring diagram of HMDB Super Class, lipids and lipid-like molecules showed the highest enrichment level (Fig. 1D). The metabolic pathway enrichment and clustering analysis from the KEGG database showed 27 pathways were enriched with metabolites of interest which distinguished the HSIL and adjacent tissues (Fig. 1E). Among these pathways, signal transduction in environmental information processing, translation in genetic information processing, infectious disease-parasitic in human diseases, lipid metabolism in metabolism, and immune system in organismal systems showed the highest enrichment level (Fig. 1E). The circos-diagram showed the differential metabolites in classification, the abundance of each group, VIP score and correlation of each metabolite (Fig. 1F). There were 5 metabolites of interest in lipid metabolism, of which 25-hydroxycholesterol (Alignment ID: POS9268) was significantly upregulated in HSIL tissues compared with adjacent tissues (Fig. 1G).

### The oxysterol 25-HC increases intercellular Fe (II) and enhances lipid peroxidation

**Fig. 2:**
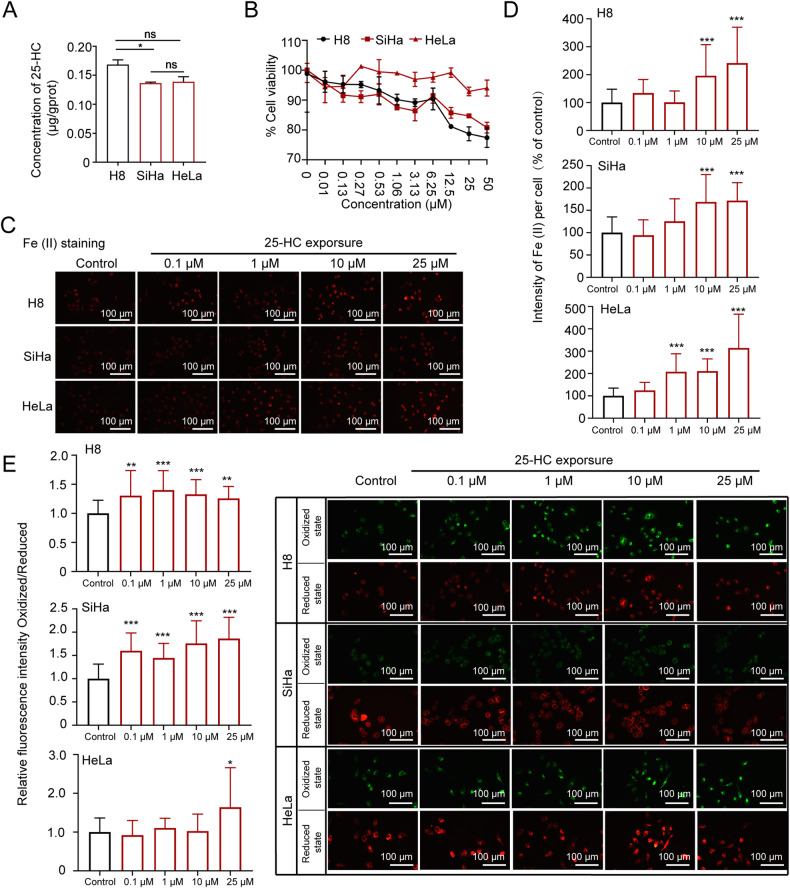
The 25-HC regulates Fe (II) and lipid peroxidation. **A** Concentration of 25-HC in H8 cells, SiHa cells, and HeLa cells was detected with an Elisa Assay Kit. All data are from three independent experiments. The data are presented as the mean ± SD values (*n* ≥ 3). **B** The H8 cells, SiHa cells, and HeLa cells were treated with 25-HC at indicated concentration for 24 h. The cell viability was detected with a CCK8 biochemical detection assay (*n* = 3). **C**, **D** The H8 cells, SiHa cells and HeLa cells were treated with 25-HC at indicated concentration for 24 h. Then they were fixed and stained with FerroOrange (red) to identify Fe (II). Scale bar, 100 μm. **E** The H8 cells, SiHa cells and HeLa cells were treated with 25-HC at indicated concentration for 24 h. Then they were fixed and stained with BDP 581/591 C11 to identify oxidized state of lipid peroxide (green) and reduced state of lipid peroxide (red). Scale bar, 100 μm. All data are from three independent experiments. The data are presented as the mean ± SD values (*n* ≥ 3). *P < 0.05; **P < 0.01, ***P < 0.001, ns: not significant.

To investigate the potential role of oxysterol 25-HC in the progress of HSIL-to-CSCC transition, three different cell lines - H8 cell lines (immortalized normal cervical epithelial cells), SiHa cell lines (cervical epithelial cells from squamous cell carcinoma patients infected with HPV16), and HeLa cell lines (cervical epithelial cells from adenocarcinoma patients infected with HPV18) were used. We first assessed the levels of 25-HC in the three cell lines with an ELISA assay, found that the 25-HC levels in SiHa cells were much lower than that in H8 cells (Fig. 2A). This result suggested that 25-HC is not required in CSCC cells. We thus examined the effect of 25-HC on cell viability in these three cell lines and observed a decrease of viability in H8 cells and SiHa cells with increasing concentrations of 25-HC, while HeLa cells exhibited greater tolerance (Fig. 2B). Furthermore, we utilized a fluorescence probe (FerroOrange) to label intracellular Fe (II) and found an increase of fluorescence intensity in all three cell lines following treatment with 25-HC, as compared to untreated cells (Fig. 2C). Notably, the increase in Fe (II) intensity was significant in H8 and SiHa cells at concentrations up to 10 μM, whereas only 1 μM of 25-HC was sufficient to significantly increase Fe (II) intensity in HeLa cells (Fig. 2D). These results strongly indicated that 25-HC had the ability to elevate intercellular Fe (II) levels. We next assessed the lipid peroxide levels in 25-HC treated cells with BDP 581/591 C11 fluorescence probe, which specifically labels oxidized lipid (green fluorescence) and reduced lipid (red fluorescence). Our findings revealed that 25-HC, at a wide range of concentrations (0.1–25 μM), enhanced lipid peroxidation in H8 and SiHa cells. However, in HeLa cells, lipid peroxidation was only enhanced when the concentration of 25-HC reached 25 μM (Fig. 2E). These results indicated that 25-HC might promote ferroptosis.

### The oxysterol 25-HC enhances ferritinophagy

Ferritinophagy is a biological process of intracellular iron intake, free iron storage and release, which is related to the initiation of ferroptosis. In this process, the iron-loaded Serotransferrin (TF) binds to TFRC and forms a TF-TFRC complex for iron uptake [19, 20], then this complex releases iron into the cytoplasm [21, 22]. The free iron is often stored as a ferritin-iron complex. When autophagy is activated, this complex is mediated by NCOA4 for ferritin-iron release in lysosomes.

**Fig. 3:**
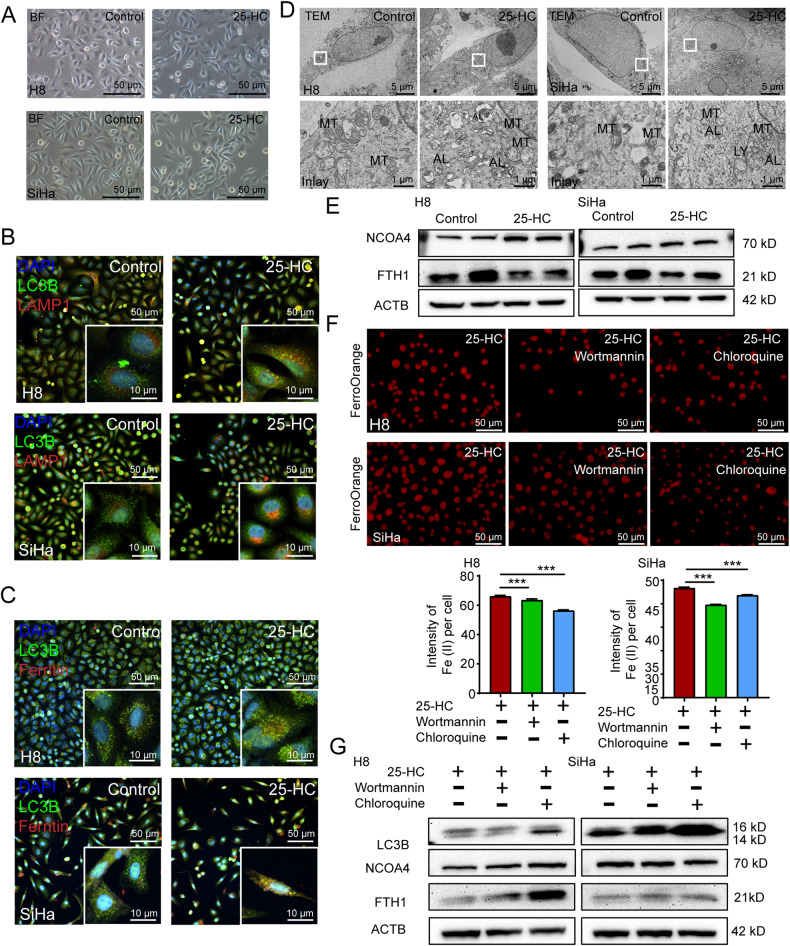
Autophagy is required in the progress of ferritinophagy induced by 25-HC. **A** Bright field images of the H8 cells and SiHa cells treated with or without 25-HC (10 μM). Scale bar, 50 μm. **B** The H8 cells and SiHa cells were treated with or without 25-HC for 24 h. Then they were fixed and immune-stained with anti-LC3 (green) and anti-LAMP1 (red). Scale bar, 50 μm (inserts, 10 μm). **C** The H8 cells and SiHa cells were treated with or without 25-HC for 24 h. Then they were fixed and immune-stained with anti-LC3 (green) and anti-FTH1 (red). Scale bar, 50 μm (inserts, 10 μm). **D** TEM images of ultrastructure in the H8 cells and SiHa cells treated with or without 25-HC. MT, mitochondria; AL, autolysosome; LY, lysosome; Scale bar, 5 μm (inserts, 1 μm). **E** Lysates of the H8 cells and SiHa cells treated with or without 25-HC for 24 h were immunoblotted for NCOA4, FTH1 and ACTB (*n* = 3). The data are normalized to the ACTB control. **F** The H8 cells and SiHa cells were treated with wortmannin or chloroquine for 1 h and continued cultured adding 25-HC for 24 h. Then they were fixed and stained with FerroOrange (red) to identify Fe (II). Scale bar, 50 μm. **G** Lysates of the H8 cells and SiHa cells treated with wortmannin or chloroquine were immunoblotted for LC3B, NCOA4, FTH1 and ACTB (*n* = 3). The data are normalized to the ACTB control. All data are from three independent experiments. The data are presented as the mean ± SD values (*n* ≥ 3). ***P < 0.001.

To further confirm whether 25-HC affected the progress of ferritinophagy, we treated the H8 cells and SiHa cells with 10 μmol 25-HC for 24 h, but did not find any change in cellular morphology (Fig. 3A). The immunofluorescent staining results showed an increased co-localization of LC3B proteins (an autophagosomes marker) with LAMP1 proteins (a lysosomes marker) in H8 cells and SiHa cells treated with 25-HC, indicating that 25-HC promoted autophagy (Fig. 3B). Moreover, as shown by fluorescence microscopy, we found an increased co-localization of LC3B proteins with FTH1 proteins (ferritin) in the cells treated with 25-HC, suggesting that 25-HC promoted ferritinophagy (Fig. 3C). To further investigate the effect of 25-HC on ferritinophagy, the ultrastructure of H8 cells and SiHa cells grown with or without 25-HC loading was observed using TEM (Fig. 3D). Consistent with the characteristics of ferroptosis, the quantity of mitochondrial cristae was found to be reduced in the 25-HC treated cells compared to their untreated counterparts. Additionally, there were more autolysosomes in the 25-HC treated cells (Fig. 3D). The western blotting results also showed an increase of NCOA4 and a decrease of FTH1 in the H8 cells treated with 25-HC compared to those in their untreated cells (Fig. 3E). Similar results were observed in SiHa cells, with a slight increase in NCOA4 expression upon 25-HC treatment. These results indicated that 25-HC promoted ferritinophagy by upregulating NCOA4 expression.

We then verified whether inhibiting autophagy could rescue. In this study, two autophagy inhibitors (wortmannin and chloroquine) were selected for this study. Wortmannin inhibits autophagosome formation; while chloroquine blocks the degradation of autophagosome in lysosome. We labeled intracellular Fe (II) with a fluorescence probe (FerroOrange) to further verify whether inhibiting autophagy could reduce Fe (II) level. The results showed that both wortmannin and chloroquine decreased the fluorescence intensity of Fe (II) in H8 cells and SiHa cells, indicating that inhibiting autophagy can repress ferritinophagy induced by 25-HC (Fig. 3F). The western blotting results showed that both inhibitors increased FTH1 levels in H8 cells and SiHa cells, while NCOA4 expression remained unchanged (Fig. 3G). These findings suggested that inhibiting autophagy could rescue the degradation of ferritin induced by 25-HC.

In summary, these results suggested that 25-HC increases intracellular Fe (II) levels by enhancing ferritinophagy in an autophagy-dependent manner.

### The oxysterol 25-HC improves cell sensitivity to ferroptosis inducer

**Fig. 4:**
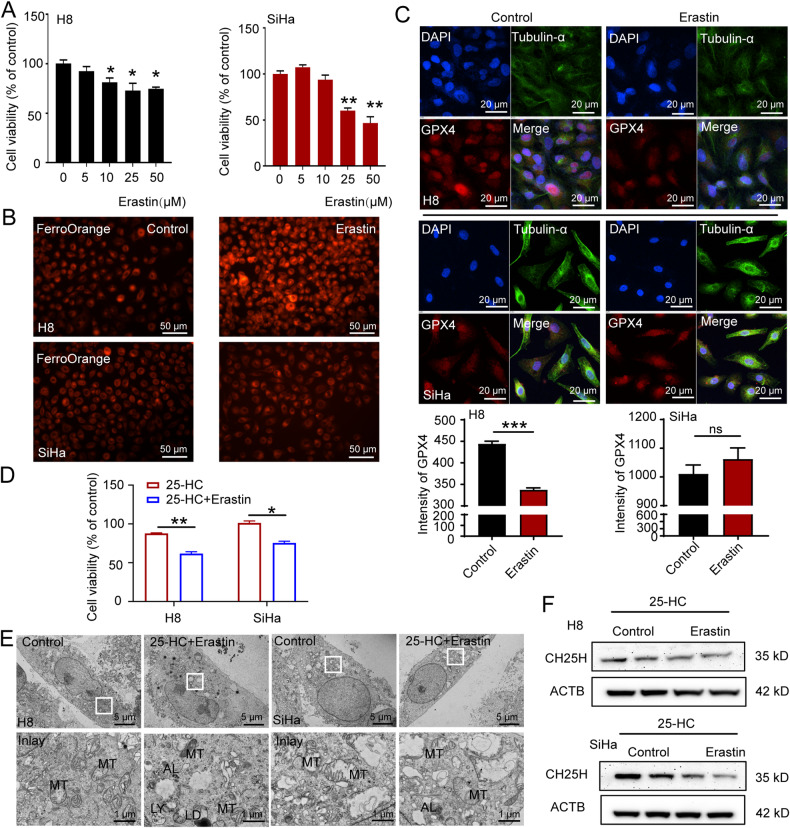
25-HC increases cell sensitivity of SiHa cells to ferroptosis. **A** The H8 cells and SiHa cells treated with erastin at indicated concentration for 24 h. The cell viability was detected with a CCK8 biochemical detection assay (*n* = 3). **B** The H8 cells and SiHa cells were treated with erastin for 24 h. Then they were fixed and stained with FerroOrange (red) to identify Fe (II). Scale bar, 50 μm. **C** The H8 cells and SiHa cells were treated with or without erastin for 24 h. Then they were fixed and immune-stained with anti-Tubulin-α (green) and anti-GPX4 (red). The nucleus was stained with DAPI (blue). Scale bar, 20 μm. **D** The H8 cells and SiHa cells treated with or without 25-HC for 24 h, then they were treated with or without erastin for another 24 h. Cell viability was detected with a CCK8 biochemical detection assay (*n* = 3). **E** TEM images of ultrastructure in the H8 cells and SiHa cells. MT, mitochondria; AL, autolysosome; LY, lysosome; LD, lipid droplet. Scale bar, 5 μm (inserts, 1 μm). **F** Lysates of the H8 cells and SiHa cells were immunoblotted for CH25H and ACTB (*n* = 3). The data are normalized to the ACTB control. All data are from three independent experiments. The data are presented as the mean ± SD values (*n* ≥ 3). *P < 0.05, **P < 0.01, ***P < 0.001, ns: not significant.

We have previously reported that ferroptosis occurred through the SIL stage, and persistent ferroptosis caused anti-ferroptosis in the CSCC stage and promoted oncogenes expression [17]. During the transition from SIL to CSCC, the key ferroptosis-related gene GPX4, was up-regulated to counteract the increased lipid peroxidation. To simulate the ferroptosis in HSIL tissues, we treated the H8 cells and SiHa cells with erastin, a classical ferroptosis inducer, and evaluated their sensitivity to ferroptosis. As shown in Fig. 4A, the cell viability was significantly decreased under the 10 μM erastin treatment in H8 cells, but not in SiHa cells. Additionally, upon treatment with 10 μM erastin, there was a significant increase of fluorescence intensity of Fe (II) in H8 cells, whereas this increase was slight in SiHa cells (Fig. 4B). Furthermore, we performed the immunofluorescent staining of GPX4, and found that 10 μM erastin significantly reduced GPX4 in H8 cells but had no significant effect on SiHa cells (Fig. 4C). These findings suggested that SiHa cells were less sensitive to ferroptosis induced by erastin than H8 cells. We next verified whether 25-HC could improve cell sensitivity of SiHa cells to erastin. As shown in Fig. 4D, the cell viability of both H8 cells and SiHa cells was significantly decreased in the 25-HC/erastin treatment groups compared to their respective single 25-HC treatment groups, suggesting that 25-HC could enhance sensitivity to ferroptosis. To describe the characteristic of ferroptosis at subcellular organelles, we performed TEM observation. We found that the quantity of mitochondrial cristae was reduced in the 25-HC/erastin-treated cells compared to their untreated counterparts (Fig. 4E). However, we found that the expression levels of CH25H were inhibited by erastin in H8 cells and SiHa cells (Fig. 4F), indicating that further induction of ferroptosis might lead to an inhibition of 25-HC and even cause anti-ferritinophagy.

### Maintaining of 25-HC level in CSCC cells increased lipid peroxidation

**Fig. 5:**
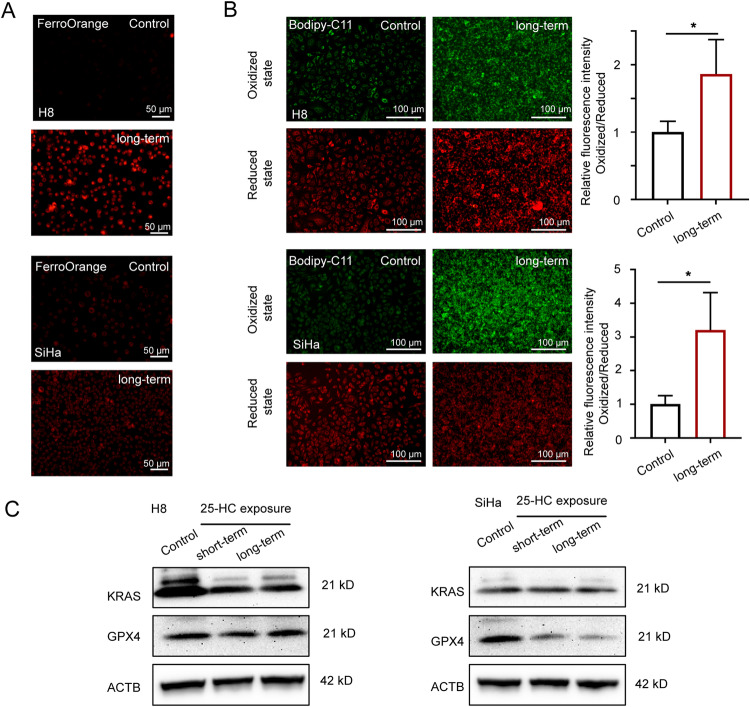
Long-term exposure to 25-HC enhances lipid peroxidation. **A** The H8 cells and SiHa cells were treated with 25-HC for 9 days (long-term). Then they were fixed and stained with FerroOrange (red) to identify Fe (II). Scale bar, 50 μm. **B** The H8 cells and SiHa cells were stained with BDP 581/591 C11 to identify oxidized state of lipid peroxide (green) and reduced state of lipid peroxide (red). Scale bar, 100 μm. **C** The H8 cells and SiHa cells were treated with 25-HC for 24 h (short-term) or 9 days (long-term). Lysates of the H8 cells and SiHa cells were immunoblotted for GPX4, KRAS, and ACTB (*n* = 3). The data are normalized to the ACTB control. All data are from three independent experiments. The data are presented as the mean ± SD values (*n* ≥ 3). *P < 0.05.

Our above data showed that ferroptosis might inhibit endogenous 25-HC generation, this reminded us to maintain intracellular 25-HC level by exogenous 25-HC treatment. Because the transition from SIL to CSCC is often a lengthy process, evaluating the effect of short-term exposure (24 h) to 25-HC alone is insufficient. It is necessary to determine whether prolonging exposure time could result in a similar trend. We verified the Fe (II) level in the short-term groups (24 h) and long-term groups (9 days), found that the fluorescence intensity of Fe (II) was higher in the long-term groups than that in control groups (Fig. 5A). Moreover, we observed a slight increase of lipid peroxidation in the long-term group of H8 cells compared to the control group, while lipid peroxidation was significantly enhanced in the long-term group of SiHa cells compared to the control group (Fig. 5B). These results suggested that maintaining 25-HC level was more detrimental to CSCC cells. In addition, the western blotting results of SiHa cells demonstrated that GPX4 was decreased in both the short-term group and the long-term group compared to the control group (Fig. 5C). Additionally, we found that both short-term exposure and long-term exposure inhibited KRAS expression in H8 cell, and had little influence of KRAS expression in the SiHa cells (Fig. 5C), indicating maintaining 25-HC could suppress the transition of normal cells to CSCC cells.

### CH25H expression level and its metabolite 25-HC level are negatively correlated with the progression of SIL development

**Fig. 6:**
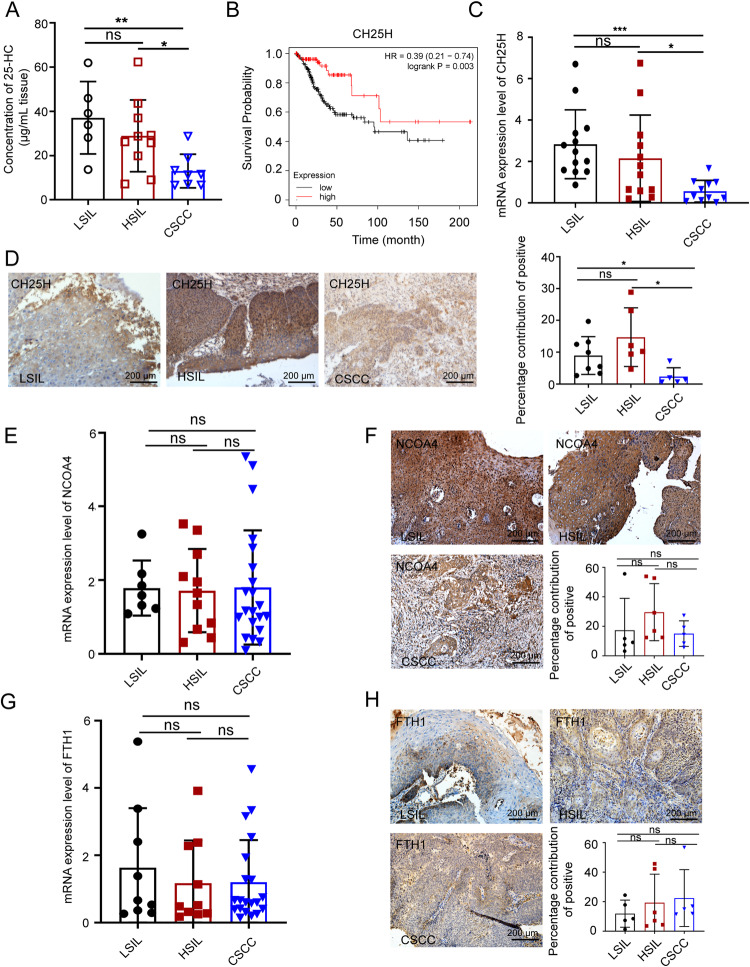
25-HC is associated with SIL development and CSCC. **A** Concentration of 25-HC in LSIL, HSIL, and CSCC tissues was detected with an Elisa Assay Kit. **B** Survival probability analysis of the expression of CH25H in patients with CSCC using Kaplan–Meier Plotter. Black line indicates low expression and red line indicates high expression. **C** qPCR was used to analyze the mRNA levels of CH25H in LSIL, HSIL, and CSCC tissues. **D** Tissue sections in LSIL, HSIL and CSCC were immunostained with anti-CH25H antibody using IHC. Scale bar, 200 μm. **E** qPCR was used to analyze the mRNA levels of NCOA4 in LSIL, HSIL, and CSCC tissues. **F** Tissue sections in LSIL, HSIL, and CSCC were immunostained with anti- NCOA4 antibody using IHC. Scale bar, 200 μm. **G** qPCR was used to analyze the mRNA levels of FTH1 in LSIL, HSIL, and CSCC tissues. **H** Tissue sections in LSIL, HSIL, and CSCC were immunostained with anti-FTH1 antibody using IHC. Scale bar, 200 μm. All data are from three independent experiments. The data are presented as the mean ± SD values (*n* ≥ 3). *P < 0.05, **P < 0.01, ***P < 0.001, ns: not significant.

We have previously reported that persistent ferroptosis caused anti-ferroptosis in CSCC stage [17], and our in vitro data showed that ferroptosis inhibited endogenous 25-HC generation by down-regulating CH25H expression. To verify this hypothesis in vivo, 24 cervical specimens including LSIL, HSIL, and CSCC were used to detect 25-HC level with an Elisa assay. We found that the levels of 25-HC in LSILs and HSILs were higher than those in CSCCs, but there was no significant difference between LSILs and HSILs (Fig. 6A). This result suggested that 25-HC generation was reduced through HSIL-to-CSCC transition. Consequently, we evaluated cholesterol 25-hydroxylase (CH25H), the gene encoding enzymes involved in oxysterol 25-HC biosynthesis, using Kaplan–Meier Plotter (http://kmplot.com/). We found that among all the CSCC patients, those with the high expression level of CH25H in tumor tissue had a higher survival probability, indicating that CH25H mRNA expression is positively correlated with the survival probability of CSCC patients (Fig. 6B). To further validate the expression level of CH25H in LSILs, HSILs, and CSCCs, over 50 cervical specimens, including LSIL, HSIL, CSCC were collected. The qPCR results showed that the mRNA expression levels of CH25H in LSILs and HSILs were significantly higher than that in CSCCs, but there was no significant difference between LSILs and HSILs (Fig. 6C). We also performed IHC on tissue sections of LSIL, HSIL, and CSCC to evaluate the protein expression levels of CH25H. The results demonstrated that the protein expression levels of CH25H were higher in LSILs and HSILs than that in CSCCs (Fig. 6D), indicating altered CH25H expression during the transition from HSIL to CSCC. These results suggested that CH25H expression and the generation of its metabolite 25-HC were both inhibited through SIL-to-CSCC transition.

We next validated the expression levels of NCOA4 and FTH1, the most important genes of ferritinophagy, in LSILs, HSILs, and CSCCs. As shown in Fig. 6E, G, the mRNA expression levels of NCOA4 and FTH1 was not significantly changed through HSIL to CSCC. IHC results showed that NCOA4 had a lighter staining in LSILs and HSILs compared to CSCCs (Fig. 6F), while FTH1 had a deeper staining in HSILs and CSCCs compared to LSILs (Fig. 6H). Collectively, these results indicated that ferritinophagy was inhibited during the CSCC stage.

Taken together, 25-HC promoted ferritinophagy through an autophagy-dependent manner during SIL development progression; persistent ferroptosis induced anti-ferritinophagy effect by inhibiting 25-HC generation.

## Discussion

In this study, we have reported an association between 25-HC levels and the development of SIL as well as the emergence of CSCC. It is now generally accepted that high-risk HPVs gain access to the basal cells of the basement membrane through injured sites in the cervical epithelium, leading to productive viral replication early in the LSIL stage [1, 23]. One important effect of 25-HC is its anti-viral function. Previous research has shown that 25-HC is capable of inhibiting the replication of HPV16 in HeLa cell lines (cervix epithelial cells from adenocarcinoma patients) [9, 10]. Additionally, 25-HC acts as a pro-inflammatory mediator. Inflammation occurs in response to harmful stimuli in the body’s tissues [24], and 25-HC has been found to be associated with an inflammatory response in various organs. For example, a study on lung inflammation revealed elevated levels of 25-HC following the emergence of lipopolysaccharide-induced acute lung inflammation [25]. In immune cells, 25-HC has a negative correlation with IL-10 secretion from type 1 regulatory T cells [26]. Furthermore, in primary trophoblasts, it promotes the production of proinflammatory cytokines in a concentration-dependent manner [27]. In our study, we observed increased CH25H expression and elevated 25-HC levels in LSILs, suggesting that endogenous synthesis of 25-HC is upregulated during the early stages of HPV infection and viral replication. This increase of 25-HC could potentially be a defensive mechanism in response to HPV infection, leading us to consider the possibility of maintaining high 25-HC levels to inhibit the development of CSCC.

Here, our study reveals the relationship between 25-HC and the regulation of iron homeostasis in cervical tissues. The induction of ferroptosis can be divided into two main pathway: the extrinsic pathway, which is related to cystine/glutamic acid transport (system xc^−^) and iron uptake (including TFRC, NCOA4, ferritin components); the intrinsic pathway, which is related to antioxidant enzyme (including unsaturation fatty acid pathway, MVA pathway related to cholesterol biosynthesis, transsulfuration progress, AIFM2-CoQ10 axis, and GCH1-BH4 pathway, et al.) [28]. Oxysterols, which are cholesterol metabolites, have been reported to regulate ferroptosis. For instance, chronic exposure to 27-hydroxycholesterol increases the antioxidant enzyme GPX4 and inhibits ferroptosis [29]. 25-HC is a natural ligand of several cholesterol transport proteins, such as oxysterol binding protein-like 2 (OSBPL2) and NPC intracellular cholesterol transporter 1 (NPC1). In HeLa cells, 25-HC is reported to inhibit OSBPL2 expression [30]. The lack of OSBPL2 may increase cholesterol biosynthesis progress and generates more ROS [31, 32]. Lack of NPC1 induces ferritinophagy in auditory cells [33]. Previous studies have reported that 25-HC promotes inflammatory response, autophagy, and increases ROS levels. However, the effect of oxysterol 25-HC on ferroptosis has not been reported in SILs and CSCCs. Our results demonstrate that 25-HC promotes ferritinophagy at the early stage after HPV infection and viral replication.

In this study, we aimed to demonstrate the effect of ferroptosis on the levels of 25-HC. The endogenous 25-HC level is primarily regulated through two ways. The first pathway involves 25-HC synthesis, where cholesterol is converted to 25-HC by the enzyme CH25H [8]. The second pathway is 25-HC metabolism, where extrahepatic 25-HC can be stored as lipid droplet by esterification or transferred into liver for further catabolism [34]. In our study, we provided evidence showing that the inducer of ferroptosis, erastin, increased lipid droplet formation and inhibited the expression of CH25H in H8 cell lines during 25-HC treatment. Although we did not observe lipid droplets under TEM observation in SiHa cells, erastin still reduced the expression of CH25H in these cells. Our findings suggest that inhibiting the production of endogenous 25-HC is one possible approach to counteracting ferroptosis. Moreover, our data also supports this view, as we observed similar trends in CH25H expression and 25-HC levels during the transition from HSIL to CSCC.

In addition, we conducted further analysis on the impact of prolonged exposure to 25-HC in CSCC cells. Our previous study indicated that persistent and mild ferroptosis facilitated an antiferroptotic effect by upregulating GPX4 in CSCC cells. Therefore, solely relying on the inherent ferroptotic effect or simple induction of ferroptosis may not be sufficient to eliminate CSCC cells. This prompted us to consider that maintaining a 25-HC level could potentially promote ferroptosis in CSCC cells. In our study, we demonstrated that long-term exposure to 25-HC suppressed GPX4 expression in CSCC cells. Therefore, maintaining a long-term 25-HC level holds promising therapeutic potential for suppressing tumor growth in CSCC.

In conclusion, our results suggested that 25-HC enhances ferritinophagy and promotes ferroptosis during SIL development progress; persistent ferroptosis reduces the level of 25-HC in cancer cells by inhibiting its synthesis, ultimately promoting further development of CSCC cells. Through the study of 25-HC’s function, we provide a theoretical basis for further better understanding the regulatory mechanism in the transition from SIL to CSCC.

### Limitations of the study

The data reported here address the regulation mechanism of 25-HC-induced ferritinophagy in the SIL stage and show that persistent ferroptosis can reduce the 25-HC level by inhibiting 25-HC synthesis in the CSCC stage. However, we acknowledge that the present study has several limitations, the detailed mechanism of ferroptosis affecting 25-HC remains to be determined. Future studies will focus on relationship of ferroptosis with 25-HC and other oxysterol in the future.

## Methods and materials

### Chemicals and antibodies

FerroOrange (#F374), ROS Assay Kit -Photo-oxidation Resistant DCFH-DA (#R253) and Lipid Peroxidation Probe-BDP 581/591 C11 (#L267) were purchased from DOJINDO LABORATORIES (Kumamoto, Japan). Antibodies against GPX4 (#A1933), NCOA4 (#A5695), ACTB (#AC026), FTH1 (#A19544), LC3 (#A17424), KRAS (#A12704) as well as secondary HRP-conjugated Goat Anti-Rabbit IgG (#AS014), secondary HRP-conjugated Goat Anti-Mouse IgG (#AS003) were from ABclonal (Wuhan, China). Antibodies against CH25H (#ER1919-26) were from HUABIO (Hangzhou, China). Erastin (#S7242) was purchased from Selleckchem (Shanghai, China). Chloroquine (#HY-17589A) and wortmannin (#HY-10197) were purchased from MCE (Shanghai, China). 25-HC (#H1015) was purchased from Sigma-Aldrich (MO, USA).

### Cell culture and treatment

The H8, SiHa, and HeLa cells were cultured in Dulbecco’s modified Eagle’s medium (DMEM, Biochannel, China) supplemented with 10% fetal bovine serum (FBS, ExCell, China) in a humidified atmosphere containing 5% CO_2_ at 37 °C. For 25-HC treatment, 25-HC (#H1015) powder was firstly dissolved in ethanol and then was diluted with complete medium at the indicated concentration. Then, the cells were cultured in a complete medium containing 25-HC for 24 h. For long-term treatment of 25-HC, old medium was removed and fresh complete medium containing 25-HC was added every 48 h. For wortmannin or chloroquine treatment, chloroquine (#HY-17589A) or wortmannin (#HY-10197) powder was firstly dissolved in DMSO and then was diluted with the complete medium at the indicated concentration. Secondly, the cells were cultured in complete medium containing chloroquine or wortmannin for 1 h. Then the complete medium was removed and the cells were cultured in complete medium containing chloroquine or wortmannin together with 25-HC for 24 h.

### Western blot analysis

Cells were lysed with RIPA buffer added with protease inhibitor cocktail (1:50, #P1005, Beyotime, China) for protein sample preparation. The samples were separated by SDS-PAGE (#M00666, GenScript, China) and transferred onto PVDF membranes (Merck, Germany). The membranes were blocked with a Blocking Buffer (#P0252, Beyotime, China) for 30 min and then incubated with primary antibody (used at 1:1000 dilution) overnight at 4 °C. The next day, the membranes were washed with Western Wash Buffer (#P0023C6, Beyotime, China) twice and then were incubated with secondary antibodies (used at 1:5000 dilution) for 1 h. The membranes were washed with Western Wash Buffer twice and incubated with BeyoECL kit (#P0018FS, Beyotime, China). The bands were visualized by a chemiluminescence method using ProteinSimple protein detection and analysis instruments (Bio-Techne, USA).

### Total RNA isolation and real-time fluorescence quantitative PCR analysis

The tissues were homogenized with TRIzol reagent (Invitrogen, USA) for total RNA extraction. The cDNA was obtained using a HiScript II one step RT-PCR kit (#R212-01, Vazyme, China). The quantitative real-time PCR (qRT-PCR) was performed on a StepOne Plus system (Applied Biosystems, USA) with ChamQ SYBR qPCR Master Mix (#R341-02, Vazyme, China). The primers used to amplify the specific genes are listed in Table S1.

### Immunohistochemistry

The paraffin sections were subjected to gradient ethanol dewaxing and followed by antigen repair. Then, the samples were blocked with goat serum (used at 1:10 dilution, #16210064, Gibico, USA) for 1 h at room temperature, and incubated overnight with primary antibodies (used at 1:100 dilution) at 4 °C. The next day, the samples were washed with PBST and incubated with secondary antibodies for 1 h at 37 °C. Images were acquired by microscopy.

### labile Fe (II) detection

Cells were grown on coverslips in 24-well plates overnight. Before Fe (II) staining, the FerroOrange power was first dissolved in 35 μL DMSO to prepare a stock solution. Then samples were stained in FerroOrange (used at 1:1000 dilution) in 10 mM PBS (pH 7.4) for 30 min at 37 °C in a dark chamber and imaged immediately. A fluorescent microscope was used to acquire high-quality images.

### Lipid Peroxidation detection

Cells were grown in 24-well plates overnight. Before lipid peroxidation staining, the BDP 581/591 C11 power was firstly dissolved in 20 μL DMSO to prepare stock solution. The samples were stained in BDP 581/591 C11 working solution (used at 1:1000 dilution) for 30 min at 37 °C in a dark chamber. Then the BDP 581/591 C11 working solution was removed and the cells were washed with HBSS twice. The images were acquired using a fluorescent microscope.

### Immunofluorescence

Cells were grown on coverslips in 24-well plates overnight. The samples were fixed in 10% formalin, and then were penetrated with 0.1% triton X-100 (Sigma). Then, the samples were blocked with goat serum (used at 1:10 dilution, #16210064, Gibico, USA) for 1 h at room temperature, and incubated overnight with primary antibodies (used at 1:100 dilution) at 4 °C. The next day, the samples were wash with PBST and incubated with secondary antibodies for 1 h at 37 °C: Alexa Fluor 546 donkey anti-rabbit IgG (used at 1:1000 dilution, #A10040, lifetech) or Alexa Fluor 488 donkey anti-mouse IgG (used at 1:1000 dilution, #A21202, lifetech). The nucleus was stained with DAPI (#F6057, Sigma-Aldrich, USA). Images were acquired by laser scanning confocal microscope. The Immunofluorescence colocalization analysis was performed with scatter J (a plugin for ImageJ), the presence of colocalization is defined as pearson’s coefficient value > 0.5.

### 25-HC detection

25-HC concentration was detected with an Elisa Assay Kit (#RJ23778, RENJIEBIO, China). For 25-HC detection within tissue, 1 g sample was added with 9 mL PBS (pH 7.2-7.4) and then was homogenized using a homogenizer at 4 °C. Then the extracting solution was centrifuged and used for 25-HC detection with an Elisa Assay Kit. For intracellular 25-HC detection, more than 10^6^ cells were collected and 25-HC was extracted by repeated freezing and thawing of liquid nitrogen. Then the extracting solution was used for both 25-HC detection with 25-HC Elisa Assay Kit and total protein detection with BCA protein concentration detection assay (#P0009, Beyotime, China).

### Transmission electron microscopy analysis

Cells and fresh tissues were collected and fixed with glutaraldehyde (#G1102, Servicebio, China) and the ultrastructure of the cell and tissue samples were photographed by Servicebio (Wuhan, China).

### Metabolomics analysis

Metabolomics profiling was analyzed by Shanghai Bioprofile Technology Company (Shanghai, China) using a UPLC-ESI-Q-Orbitrap-MS system (UHPLC, Shimadzu Nexera X2 LC-30AD, Shimadzu, Japan) coupled with Q-Exactive Plus (Thermo Scientific, San Jose, USA). Quality control (QC) samples were prepared by pooling aliquots of all samples that were representative of the samples under analysis, and used for data normalization. Blank samples (75%ACN in water) and QC samples were injected every six samples during acquisition. R(version:4.0.3) and R packages were used for all multivariate data analyses and modeling. Data were mean-centered using Pareto scaling. Models were built on principal component analysis (PCA), orthogonal partial least-square discriminant analysis (PLS-DA) and partial least-square discriminant analysis (OPLS-DA). All the models evaluated were tested for over fitting with methods of permutation tests. OPLS-DA allowed the determination of discriminating metabolites using the variable importance on projection (VIP). Metabolites with VIP values greater than 1.0 and *p* value less than 0.05 were considered to be statistically significant metabolites. Fold change was calculated as the logarithm of the average mass response (area) ratio between two arbitrary classes. To identify the perturbed biological pathways, the differential metabolite data were performed KEGG pathway analysis using the KEGG database (http://www.kegg.jp). KEGG enrichment analyses were carried out with Fisher’s exact test, and FDR correction for multiple testing was performed. Enriched KEGG pathways were nominally statistically significant at the p < 0.05 level.

### Sample collection

Cervical specimens including LSIL, HSIL, and CSCC tissues were collected from patients who underwent surgical operation from September 2021 to August 2022 in obstetrics and gynecology department of Jiangning Hospital. Specimens were frozen in liquid nitrogen immediately after operation and stored at −80 °C until extraction. All samples were confirmed by histopathological examination.

### Ethical statements

The clinical study was approved by the Ethics Committee of Jiangning Hospital (No. 2021-03-020-K01). All patients signed an informed consent form prior to the study.

### Statistical analysis

All data are presented as the mean ± SD. Student’s *t* test was used for comparisons between two independent sample groups, one-way analysis of variance (ANOVA) was used for single-factor comparisons among multiple groups, and two-way ANOVA was used for two-factor comparisons among multiple groups; *P* < 0.05 was regarded as significant.

### Supplementary information

Supplemental information-TABLE 1

western blots bands

## Data Availability

All data generated or analyzed during this study are included in this published article.
