# Supplementary material for: Oxysterol 25-hydroxycholesterol activation of ferritinophagy inhibits the development of squamous intraepithelial lesion of cervix in HPV-positive patients
Source: Cell Death Discov. 2024 Mar 13;10:135. doi: 10.1038/s41420-024-01899-3 (PMC10933481; doi:10.1038/s41420-024-01899-3)
Supplement: Supplementary file 1 — Supplemental information-TABLE 1 [file 41420_2024_1899_MOESM1_ESM.docx]

**Table S1** Primer sequences.

| Gene |  | primer |
| --- | --- | --- |
| ACTB (human) | Forward | 5′- GCCGAGGACTTTGATTGC-3′ |
|  | Reverse | 5′- CCTGTGTGGACTTGGGAGA-3′ |
| NCOA4 (human) | Forward | 5′-GGAAGCCTGAGAATGGC-3′ |
|  | Reverse | 5′-CACCTTTGGGCTGGTTT-3′ |
| CH25H (human) | Forward | 5′- GACCTGCATCACTCTCACTTT-3′ |
|  | Reverse | 5′- AGCAGTCCCGAGTCTTAGG-3′ |
| FTH1 (human) | Forward | 5′-AGTCGTCGGGGTTTCCT-3′ |
|  | Reverse | 5′-GAGGGTGCGGTGAAGAG-3′ |
| TFRC (human) | Forward | 5′- ACTTCACCGGCACCATC-3′ |
|  | Reverse | 5′- ATCACGCCAGACTTTGCT-3′ |
