# Supplementary material for: Oxysterol 25-hydroxycholesterol activation of ferritinophagy inhibits the development of squamous intraepithelial lesion of cervix in HPV-positive patients
Source: Cell Death Discov. 2024 Mar 13;10:135. doi: 10.1038/s41420-024-01899-3 (PMC10933481; doi:10.1038/s41420-024-01899-3)

NCOA4 related to Fig. 3e


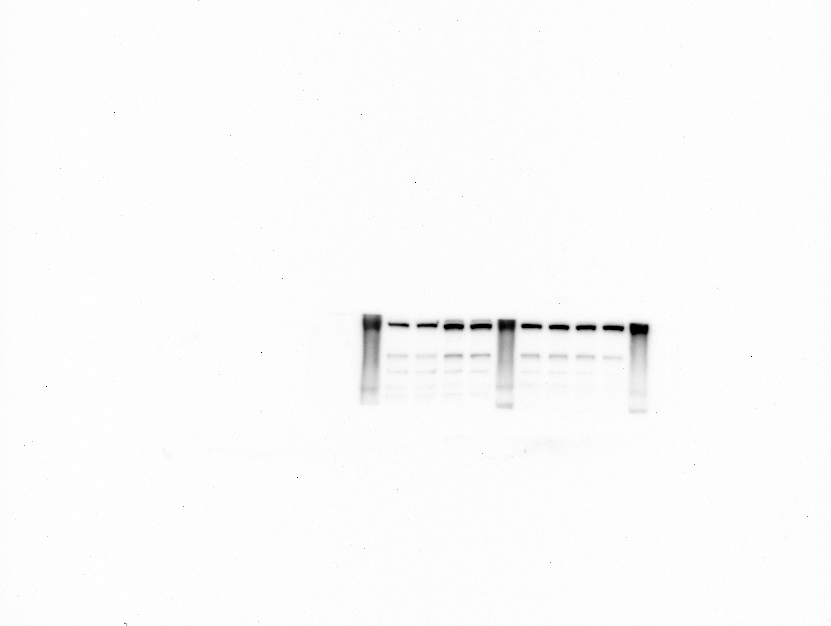


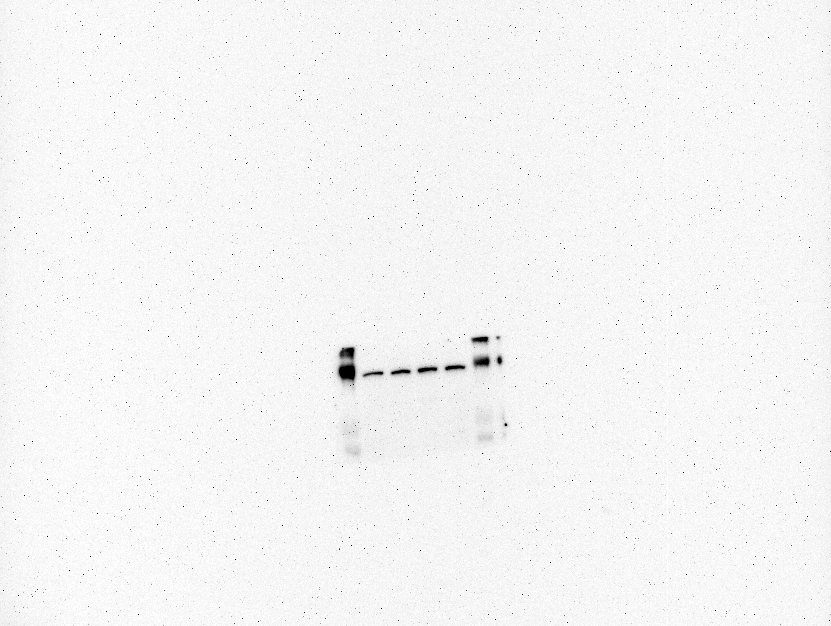


FTH1 related to Fig. 3e


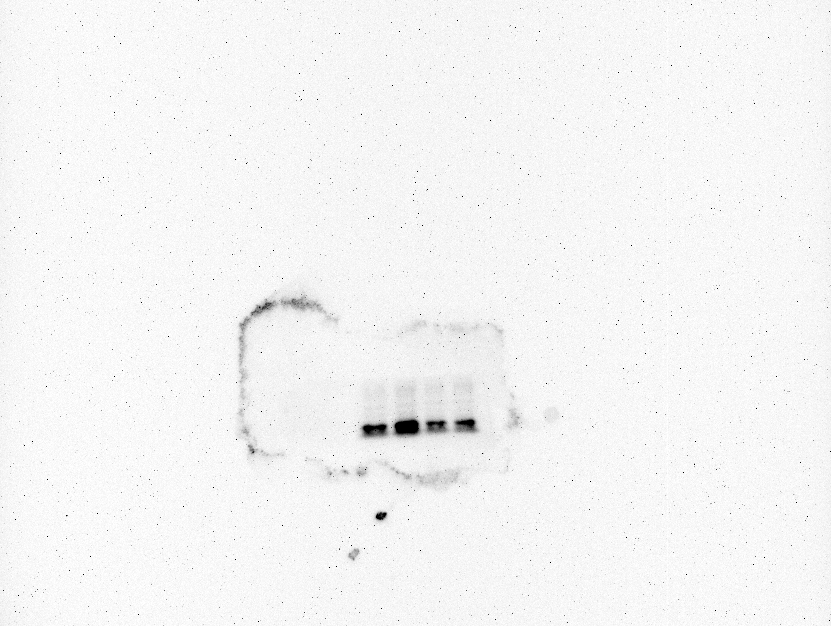


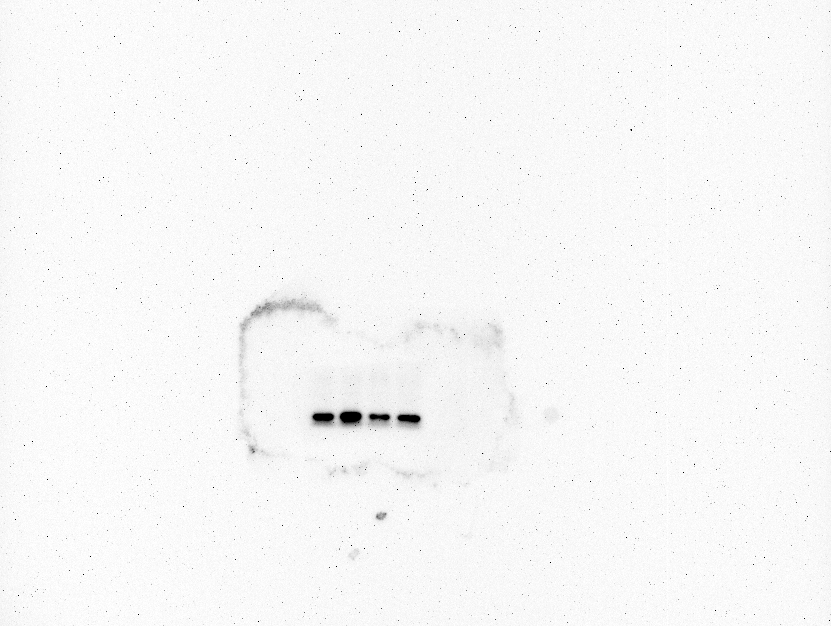


ACTB related to Fig. 3e


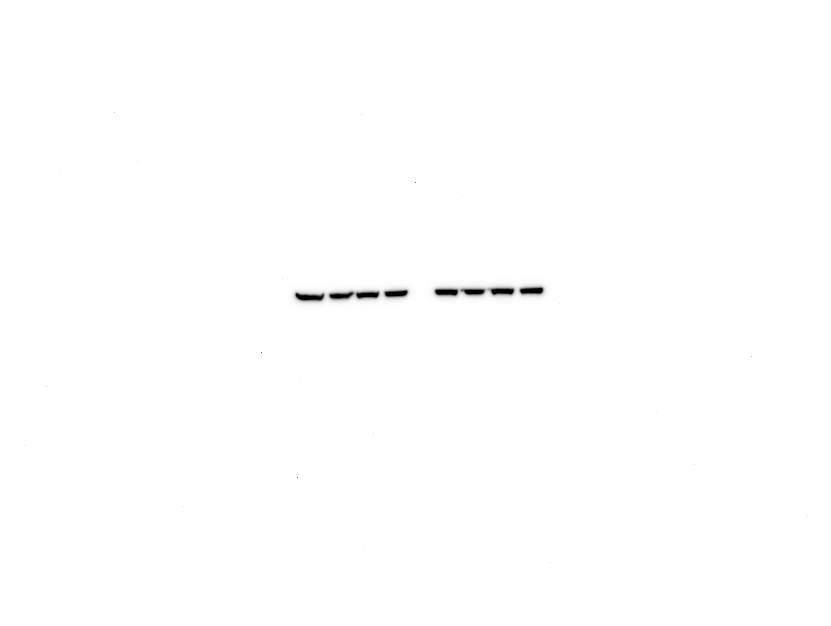


LC3B related to Fig. 3g


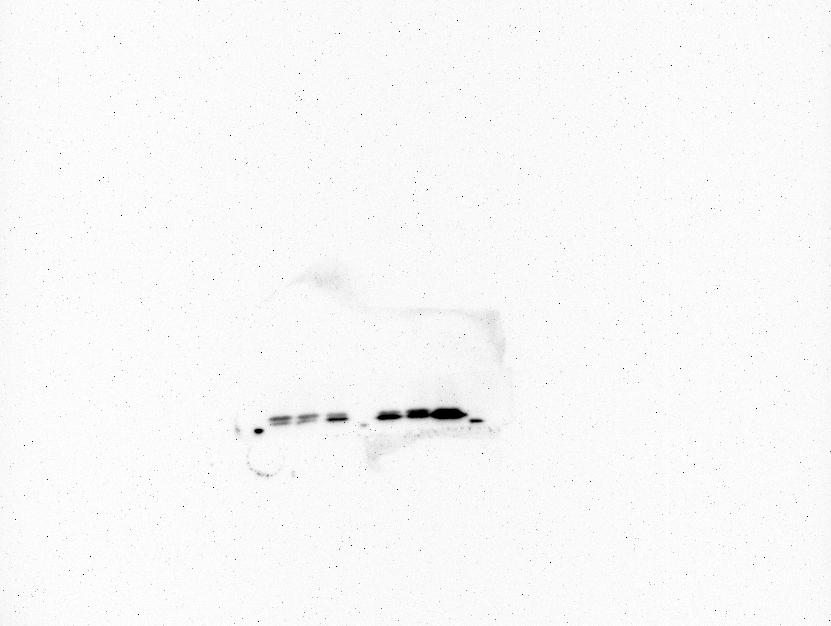


NCOA4 related to Fig. 3g


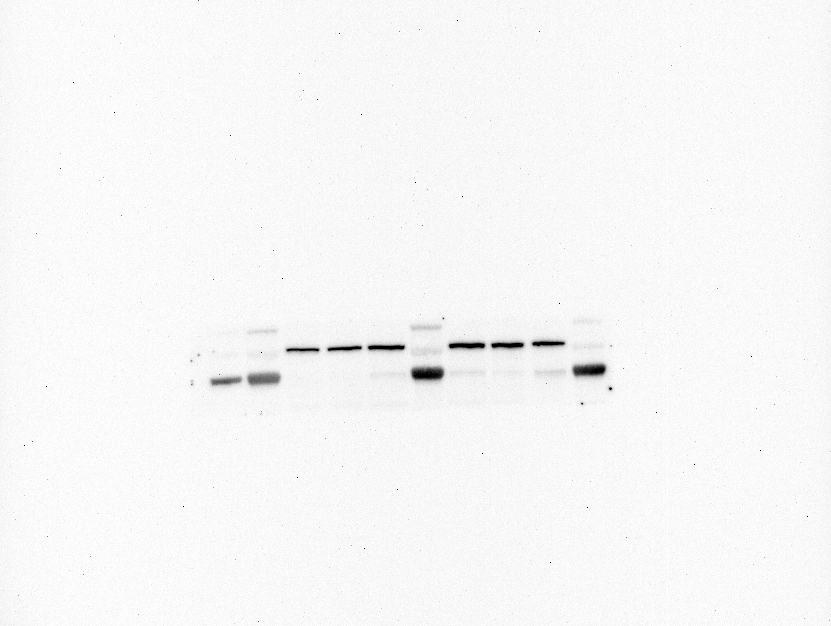


FTH1 related to Fig. 3g


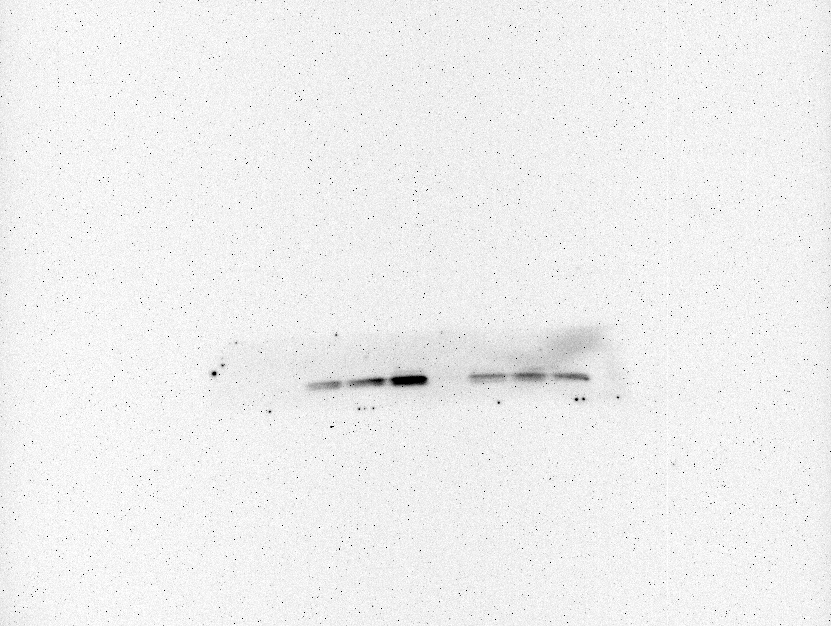


ACTB related to Fig. 3g


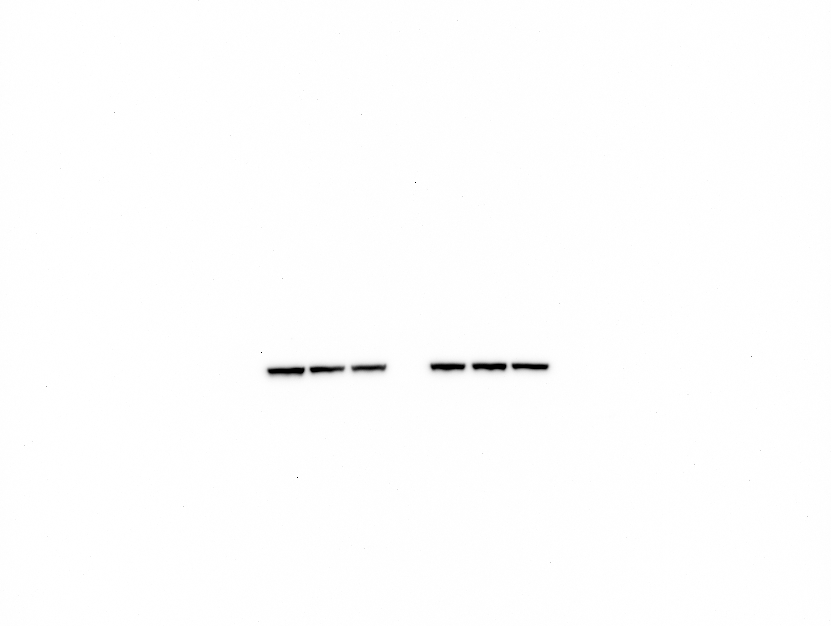


CH25H related to Fig. 4f


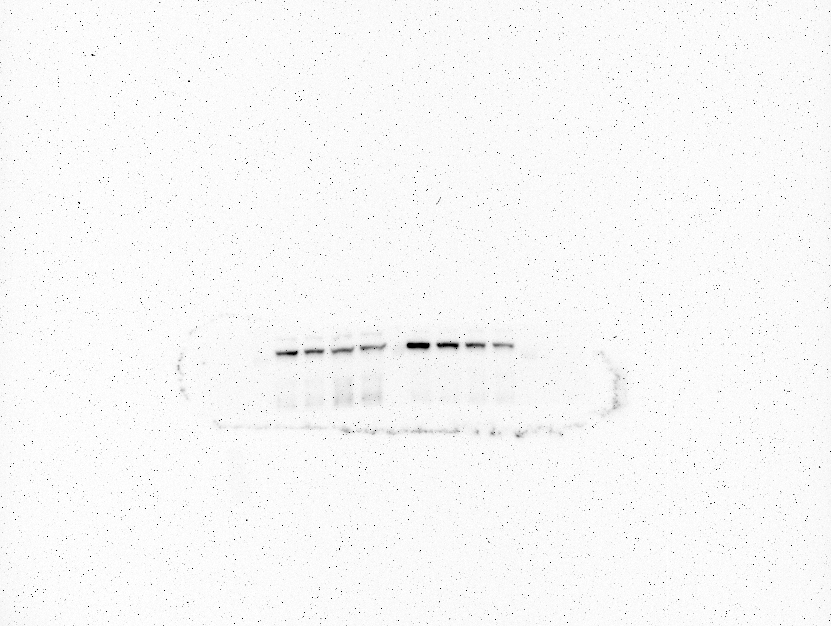


ACTB related to Fig. 4f


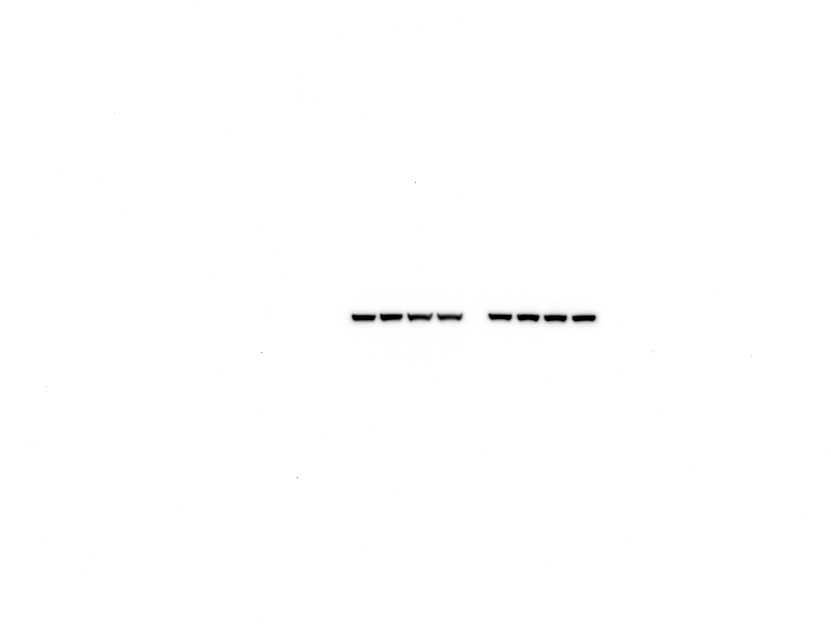


GPX4 related to Fig. 5c


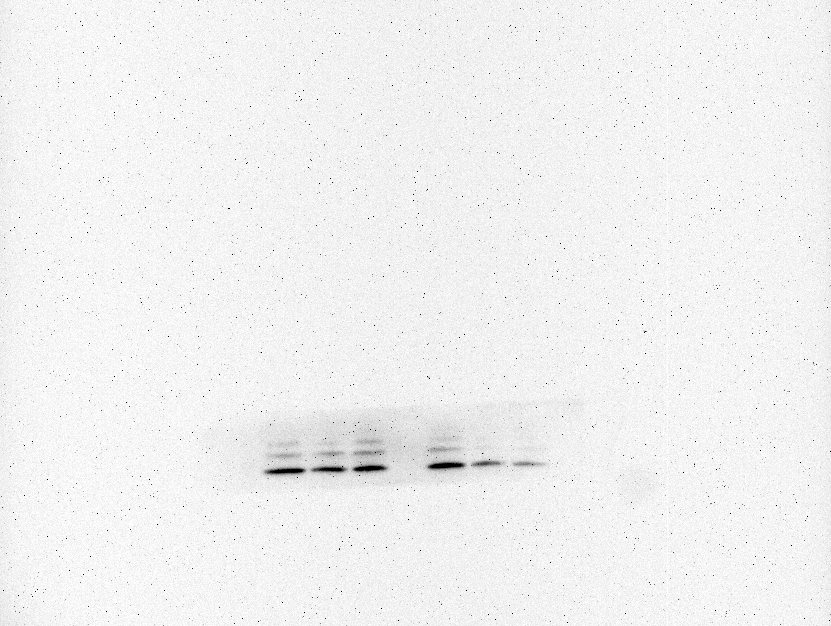


KRAS related to Fig. 5c


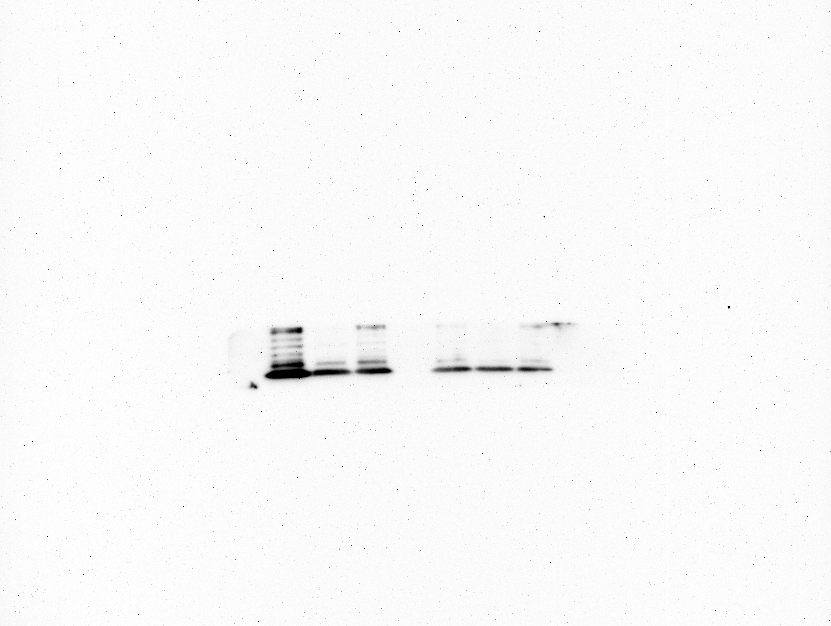


ACTB related to Fig. 5c


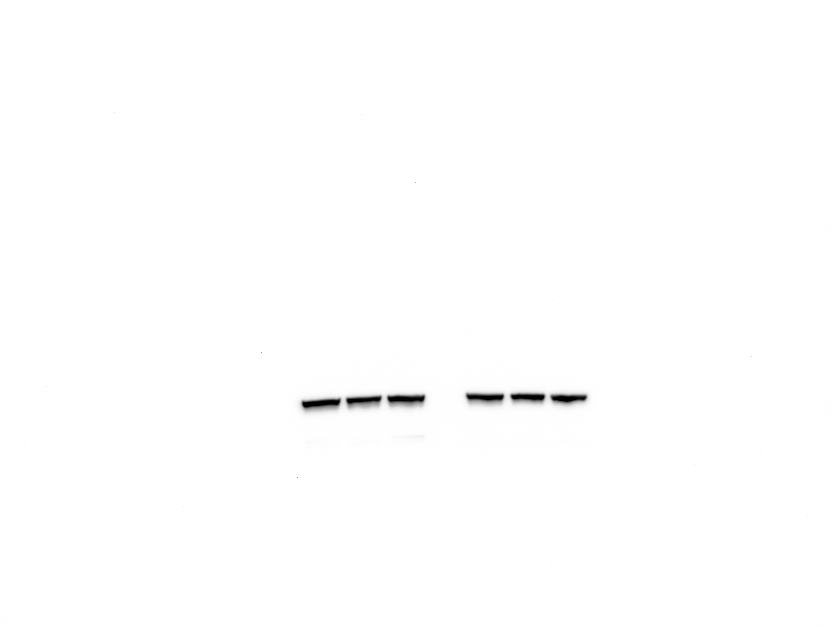

Supplement: Supplementary file 2 — western blots bands [file 41420_2024_1899_MOESM2_ESM.docx]
